# Towards People-Centred Integrated Care: From Passive Recognition to Active Co-production?

**DOI:** 10.5334/ijic.2492

**Published:** 2016-06-24

**Authors:** Nick Goodwin

**Affiliations:** Editor-in-Chief, IJIC, 10 Goldsmith Close, Bicester, UK

It has long been accepted that the concept of integrated care should be centred on the needs of service users, their families and the communities to which they belong [1]. The ability to co-ordinate care and services around people’s needs is integrated care’s ‘compelling logic’. The people-centred approach helps care systems understand that outcomes must go beyond cost-efficiencies created through new organisational and professional processes to ensure that they also improve people’s care experiences and outcomes [2]. Indeed, initiatives that employ such a people-centred narrative appear to make better progress as they have the greater potential to unite the often diverse objectives of professional, organisational and political stakeholders [3].

Yet, as discussions at the 16^th^ International Conference on Integrated Care observed, the movement towards integrated care currently takes a passive stance when it comes to the importance of engaging and empowering people [4,5]. In other words, and however well-meaning and committed, health and social care professionals most often remain paternalistic in their care and treatment of people. As a result, an opportunity is lost to harness the power of individuals, families and communities as partners in care.

The importance of co-production was recently articulated by the World Health Organisation (WHO) in their recent vote to adopt a resolution to support the *WHO Framework on Integrated People-Centred Health Services* [6]. Within this, it was argued that nothing less than a fundamental paradigm shift was required to put people and communities at the heart of the health care experience. Since people spend the majority of their time living and responding to what impacts on their own health needs, supporting people to make choices regarding healthy behaviours and improving their ability to self-care is essential (especially with underserved populations and marginalised groups).

There is, of course, good evidence to demonstrate the value of empowerment strategies to individuals, carers and families suggesting that approaches such as shared decision-making and self-management support should be better embedded in integrated care programmes than currently seems to be the case [7]. Moreover, the WHO presents evidence on how by engaging communities in health literacy, in enabling individuals to make informed choices, and in supporting citizens to understand their rights and responsibilities, so community health outcomes can be enhanced. The WHO go further to argue that the future of care therefore requires an ‘equal and reciprocal relationship’ between clinical and non-clinical professionals together with the individuals using care services, their families and communities [6].

At the present time there are few examples of how this approach to co-production with local communities can be achieved in practice. However, in this edition of IJIC, a perspective paper by Morton and Paice describes how co-production was developed with lay partners in the context of North West London, UK [8]. A key outcome of this approach was the development of an *integration toolkit* co-designed with lay partners who provided challenge, encouraged innovation, improved communication, and held the actions of other partners to account to ensure the vision and aims of the emerging integrated care system were met. Ultimately, co-production appears to have had a positive impact on the design of North West London’s integrated care system and, importantly, in how a culture of patient-centred working was established.

There can be no doubt today that the future sustainability of our care systems requires ‘full engagement’ with people and populations as a means to promote healthier lifestyles and improve wellbeing. Care systems would benefit from changing their approach so that communities, and the people living within them, become regarded as assets in achieving such goals. If integrated care as a movement for change is to work then it requires the development of a new form of partnership with people and populations. A change in perspective is indicated, therefore, from the ‘passive’ recognition that integrated care should focus on person-centred care to the ‘active’ participation, empowerment and leadership from people in the co-production of their health.
